# Genomic landscape and therapeutic implications of Anaplastic Lymphoma Kinase fusion-positive colorectal cancer

**DOI:** 10.1093/oncolo/oyag308

**Published:** 2026-08-07

**Authors:** Xuanyi Li, Faiza Yasin, Dean C Pavlick, Jeffrey S Ross, Michael Cecchini

**Affiliations:** Yale Cancer Center, New Haven, CT 06510, United States; Dana-Farber Cancer Institute, Boston, MA 02215, United States; Foundation Medicine, Inc, Boston, MA 02210, United States; Foundation Medicine, Inc, Boston, MA 02210, United States; Upstate Medical UniversitySyracuse, NY 13210, United States; Yale Cancer Center, New Haven, CT 06510, United States

**Keywords:** colorectal neoplasms, anaplastic lymphoma kinase, gene rearrangement, precision medicine, molecular targeted therapy, high-throughput nucleotide sequencing

## Abstract

**Background:**

Anaplastic lymphoma kinase (ALK) fusions are established oncogenic drivers and therapeutic targets in multiple malignancies, including non-small cell lung cancer, but are rare in colorectal cancer (CRC). Although ALK fusions in CRC have been associated with sensitivity to ALK inhibitors, their broader genomic context remains incompletely defined. We aimed to characterize the prevalence, clinicogenomic features, and therapeutic implications of ALK fusions in advanced CRC.

**Materials and Methods:**

Comprehensive genomic profiling using hybrid capture-based next-generation sequencing was performed on 56 206 advanced CRC tumors to identify alterations, including ALK fusions. Microsatellite instability (MSI)/mismatch repair (MMR) status, tumor mutational burden (TMB), genomic ancestry, COSMIC trinucleotide signatures, and homologous recombination deficiency (HRD) signatures were derived from sequencing data. PD-L1 expression was assessed by immunohistochemistry.

**Results:**

ALK fusions were identified in 63 tumors (0.1%). Compared with ALK fusion-negative CRC, ALK fusion-positive (ALKfus+) tumors demonstrated enrichment for MSI-high/MMR-deficient status and high TMB, with numerically higher PD-L1 expression. In contrast, canonical CRC driver alterations were markedly depleted, including *KRAS*, *APC*, and *PIK3CA* mutations.

**Conclusions:**

ALK fusions define a rare but biologically distinct subset of CRC characterized by frequent MSI-high/MMR-deficient status and a unique genomic landscape. These findings support comprehensive genomic profiling to identify ALK fusions in CRC, which may inform the use of ALK-directed therapies, and highlight the frequent co-occurrence of MSI-high disease within this subgroup.

Implications for Practice Anaplastic lymphoma kinase (ALK) fusions occur in a small subset of patients with colorectal cancer but identify a distinct molecular subgroup characterized by depletion of common oncogenic drivers and frequent microsatellite instability (MSI)-high/mismatch repair-deficient status. Our findings highlight the importance of comprehensive genomic profiling to identify ALK fusions and support the use of ALK-directed therapies in appropriately selected patients. The frequent co-occurrence of MSI-high disease further defines the molecular context of this subgroup and may have additional therapeutic implications, including eligibility for immune checkpoint blockade.

## Introduction

Targeted therapies have transformed the cancer treatment landscape, but their application in colorectal cancer (CRC) remains limited compared to other common malignancies such as lung and breast cancers. Precision medicine in CRC primarily involves anti-epidermal growth factor receptor (EGFR) agents for left-sided, RAS wild-type tumors, while anti-vascular endothelial growth factor (VEGF) agents are used more broadly.^1^^,^^2^ Additional targeted approaches are available for *HER2* amplification, *BRAF* V600E mutations, and *KRAS* G12C mutations, as well as for tumor-agnostic targets such as microsatellite instability high (MSI-high)/mismatch repair deficiency (dMMR), tumor mutational burden (TMB) high, and *RET* and *NTRK* fusions, yet the number of actionable targets in CRC is still relatively small.^3^ Therefore, there is a pressing need to identify additional actionable genomic alterations that will expand therapeutic opportunities for patients with CRC.

*Anaplastic lymphoma kinase* (ALK) fusions arise from chromosomal translocations, inversions, or other rearrangements that join the *ALK* gene located on chromosome 2p23 with a partner gene.^4^ The resulting fusion protein combines the promoter and oligomerization domains of the partner gene with the kinase domain of ALK, leading to constitutive receptor tyrosine kinase activity and activation of ALK signaling pathways (eg, MAPK/ERK, PI3K/AKT, JAK/STAT), which promotes cell proliferation and survival.^5^^,^^6^ ALK fusions are well-established therapeutic targets in oncology. Multiple small-molecule ATP-competitive tyrosine kinase inhibitors (TKIs) have been developed to bind the intracellular kinase domain of ALK fusion proteins, thereby preventing autophosphorylation and downstream signaling activation.^7^^,^^8^ To date, FDA-approved ALK inhibitors include crizotinib, ceritinib, alectinib, brigatinib, lorlatinib, and ensartinib, with indications primarily in ALK-positive non-small cell lung cancer (NSCLC)^9–15^ and select approvals in anaplastic large cell lymphoma (ALCL) and inflammatory myofibroblastic tumor (IMT).^16^^,^^17^ The therapeutic landscape continues to evolve with ongoing clinical development and emerging approvals for next-generation ALK inhibitors.^18^^,^^19^ However, despite the clearly defined oncogenic role of *ALK* fusions and the availability of effective ALK-targeted therapies across multiple tumor types, *ALK* fusions in CRC remain poorly characterized.

In CRC, *ALK* fusion were first described in 2012,^20^ and have since been detected by both tissue and liquid biopsies. These fusions have been associated with clinical responses to ALK inhibitors in multiple case reports and small case series.^21–24^ However, there is limited information on the incidence and clinical characteristics of *ALK* fusions in metastatic CRC. To further understand this patient population harboring a rare but potentially actionable genomic abnormality, we performed a retrospective cohort study using the Foundation Medicine database to identify the genomic and clinical features of *ALK* fusion-positive CRC.

## Methods

Approval for this study, including a waiver of informed consent, was obtained from the Western Institutional Review Board (Protocol No. 20152817). A retrospective database search of a CLIA-certified and CAP-accredited reference molecular laboratory was performed for 56 206 advanced CRC tissue samples. Clinicopathological data confirming that all cases represented clinically advanced and metastatic CRC, including patient age and sex, routine histology and immunohistochemical (IHC) staining results, and confirmation of diagnosis, were extracted from available medical records and pathology reports. One H&E slide, corresponding to the tissue that was submitted for genomic profiling, was available for each case to review morphological features.

After extraction of a minimum of 50 ng of DNA, all formalin-fixed paraffin-embedded tissue samples underwent hybrid capture-based comprehensive genomic profiling (CGP) using the FDA-approved FoundationOne® CDx platform to assess all classes of GAs including base substitutions, short insertions/deletions, copy number changes, and genomic rearrangements and fusions.^25–28^ Tumor mutational burden (TMB) was determined on at least 0.8 Mb of sequenced DNA, as previously described^29^ and was reported as mutations/Mb. Assessment of microsatellite instability (MSI) was performed from DNA sequencing at least 1500 loci, as previously described^30^ and was reported categorically as MSI-high, MSI-intermediate, or MS-stable (MSS). Single-base substitution mutational signatures were determined by assessing the trinucleotide context of detected single base substitution alterations and compared to the Sanger Catalog of Somatic Mutations in Cancer (COSMIC) signatures of mutational processes in human cancer, as previously described.^31^^,^^32^ A Homologous Recombination Deficiency signature (HRDsig) was also measured as previously described.^33^ PD-L1 expression was determined on subsets of tumors using the DAKO 22C3 assay with low positive tumor cell scoring defined as 1%-49% staining and high positive tumor cell scoring defined as ≥50% staining.

For the primary analysis, cases with ALK fusions (ALKfus+) were compared with those without ALK fusions (ALKfus-). Additional exploratory analyses were performed stratified by MSI status, with comparisons between ALKfus + and ALKfus- tumors conducted separately within the MSI-high and MSS subgroups. Fisher’s exact test and Mann-Whitney *U* test were used in the statistical comparisons of the two groups employing the Benjamini–Hochberg adjustment to correct for false discovery rate; statistical significance was defined as *P* < .05.

## Results

We identified 63 (0.1%) ALKfus + CRC cases. Clinical characteristics of ALKfus + and ALKfus- cases are summarized in Table 1. In contrast to ALKfus- CRCs, ALKfus + CRCs were older (median age 68 years vs 62 years; *P* = .012) and less likely to be male (34.9% vs 56.0%; *P* < .0001). Genomic ancestry distribution was similar between the two groups.

**Table 1.** Clinical characteristics of ALKfus + vs ALKfus**−** CRCs.

Characteristic	ALKfus + CRCs (*N* = 63)	ALKfus − CRCs (*N* = 56 143)
**Age**		
**Median**	68	62
**Range**	38-89	8-89
**Sex—no. (%)**		
**Male**	22 (34.9)	31 468 (56.0)
**Female**	41 (65.1)	24 675 (44.0)
**Genomic ancestry—no. (%)**		
**European**	48 (76.2)	40 104 (71.4)
**Admixed American**	9 (14.3)	6283 (11.2)
**African**	4 (6.3)	7035 (12.5)
**East Asian**	2 (3.2)	2215 (4.0)
**South Asian**	0 (0)	506 (0.9)

27 unique *ALK* partner breakpoint loci were identified (Table 2). The most frequent ones were *STRN-ALK* fusion (24.6%), *EML4-ALK* fusion (18.8%), *ALK* intron 19 breakpoint with no discernible partner (10.1%), and *SPTBN1-ALK* fusion (7.2%). This distribution differs from that observed in NSCLC, where *EML4-ALK* is the predominant fusion and other rearrangements are less common.^34^ When stratified by MSI status, the predominant fusion partners were similar between subgroups, with *STRN-ALK* (MSS: 22.2% [10/45] vs MSI-high: 38.9% [7/18]) and *EML4-ALK* (MSS: 17.8% [8/45] vs MSI-high: 22.2% [4/18]) remaining the most common rearrangements.

**Table 2.** Distribution of ALK partner breakpoints.

Partner breakpoint	Count	Frequency
**STRN**	17	0.246
**EML4**	13	0.188
**Intergenic space**	7	0.101
**SPTBN1**	5	0.072
**CAD**	4	0.058
**ALK**	2	0.029
**AFAP1L1**	1	0.014
**ARHGAP15**	1	0.014
**ATIC**	1	0.014
**CLIP2**	1	0.014
**CNTN4**	1	0.014
**COL5A2**	1	0.014
**DTNB**	1	0.014
**DYNC1I2**	1	0.014
**EPHA2**	1	0.014
**GTF2IRD1**	1	0.014
**ITSN2**	1	0.014
**MTA3**	1	0.014
**NPAT**	1	0.014
**PCM1**	1	0.014
**PPP1R21**	1	0.014
**RBBP8**	1	0.014
**ROCK2**	1	0.014
**SLMAP**	1	0.014
**SRSF7**	1	0.014
**TRAPPC12**	1	0.014
**TRIOBP**	1	0.014

**Figure 1.**
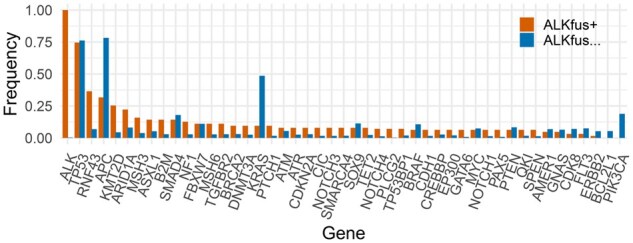
Long-tail plot of pathogenic genomic alterations (GAs) in ALK fusion-positive (ALKfus+) vs ALK fusion-negative (ALKfus−) colorectal cancer (CRC). The frequency of pathogenic GAs across genes detected by comprehensive genomic profiling is shown for ALKfus + and ALKfus − CRC tumors. Only genes altered in ≥5% of tumors in either cohort were included. Genes are ranked according to GA frequency in ALKfus + tumors.

For the primary analysis, pairwise comparisons were performed across 338 genes between ALKfus + and ALKfus- cohorts. Of these, 47 genes with alterations observed in ≥5% of tumors in either cohort were included in the analysis (Figure 1). ALKfus + CRCs demonstrated lower prevalence of several common variants in relevant CRC oncogenic pathways, including *KRAS* (9.5% vs 48.6%; *P* < .0001), *APC* (31.7% vs 78.3%; *P* < .0001), and *PIK3CA* (0.0% vs 18.9%; *P* < .0001). The actionable mutation *BRAF* V600E (6.3% vs 10.7%; NS) was also less prevalent in ALKfus + CRC. Genomic alterations in *TP53* were similar between the two groups (74.6% vs 76.2%; NS). HRDsig positivity was extremely uncommon in both groups (0.0% vs 1.7%; NS). Non-fusion *ALK* GAs were identified in 0.3% of ALKfus- CRC. A list of all pathogenic GAs that were significantly different between the two groups is shown in Table 3.

**Table 3.** Pathogenic genomic alterations differentially observed in ALKfus + vs ALKfus**−** CRC.

Genes	ALKfus+ (%)	ALKfus− (%)	*P*-value^a^
**Oncogenes**			
***ALK***	100.0	0.3	<.0001
***KRAS***	9.5	48.6	<.0001
***PIK3CA***	0.0	18.9	<.0001
***GATA6***	6.3	0.8	.008
***NOTCH1***	6.3	1.4	.028
***NOTCH3***	7.9	1.6	.012
**Tumor suppressors**			
***APC***	31.7	78.3	<.0001
***RNF43***	36.5	6.9	<.0001
***ARID1A***	22.2	8.2	.002
***SMARCA4***	7.9	1.8	.014
***CIC***	7.9	1.7	.012
***SPEN***	6.3	1.4	.027
***CDH1***	6.3	1.6	.038
***NF1***	12.7	2.9	.002
***TGFBR2***	11.1	2.9	.008
***PTCH1***	9.5	1.7	.003
**DNA repair/MMR**			
***MSH3***	15.9	3.9	.0009
***MSH6***	11.1	2.8	.008
***BRCA2***	9.5	3.0	.027
***ATR***	7.9	2.6	.044
**Epigenetic regulators**			
***KMT2D***	25.4	4.4	<.0001
***ASXL1***	14.3	5.2	.014
***DNMT3A***	9.5	2.6	.014
***TET2***	7.9	2.4	.035
**Transcription factors/post-translational regulators**			
***PAX5***	6.3	0.9	.008
***QKI***	6.3	1.7	.043

a False discovery rate (FDR) corrected using Benjamini-Hochberg procedure.

The distributions of MSI status, TMB, and PD-L1 expression across the two groups are shown in Table 4. ALKfus + CRCs were more frequently MSI-high (28.6% vs 6.0%; *P* < .0001), had higher rates of TMB ≥20 mutations/Mb (28.6% vs 6.2%; *P* < .0001), and demonstrated higher PD-L1 expression (27.3% vs 14.9%; NS), when compared with ALKfus- CRCs. COSMIC MMR-associated genomic signatures were also identified more frequently in ALKfus + CRC (31.7% vs 7.5%; *P* < .0001).^35^

**Table 4.** Immunotherapy-related biomarkers in ALKfus + vs ALKfus**−** CRC.

	ALKfus+	ALKfus−
**Microsatellite status**		
**MSS**	45 (71.4%)	50 249 (90.4%)
**MSI-L**	0	2001 (3.6%)
**MSI-H**	18 (28.6%)	3362 (6.0%)
**Tumor mutational burden (TMB)**		
**Median TMB (range)**	6.25 (0-85)	3.62 (0-648)
**TMB <10 mut/Mbp**	44 (69.8%)	51 014 (90.9%)
**TMB 10-20 mut/Mb**	1 (1.6%)	1616 (2.9%)
**TMB ≥20 mut/Mb**	18 (28.6%)	3512 (6.2%)
**PD-L1 IHC**		
**PD-L1 negative**	16 (72.7%)	18 102 (85.1%)
**PD-L1 positive**	6 (27.3%)	3167 (14.9%)

Given the enrichment of MSI-high tumors among ALKfus + CRCs, additional exploratory analyses were performed stratified by MSI status. Within the MSI-high subgroup (*n* = 3380), 18 tumors (0.5%) were ALKfus+, whereas within the MSS subgroup (*n* = 50 294), 45 tumors (0.09%) were ALKfus+. In both subgroups, no significant differences were observed between ALKfus + and ALKfus- tumors with respect to TMB or PD-L1 expression (Tables 5 and 6). Analysis of co-occurring genomic alterations demonstrated distinct patterns within the MSI-high and MSS subgroups. Within the MSI-high subgroup, ALKfus + tumors were significantly less likely to harbor BRAF and PIK3CA alterations (Table 5). Within the MSS subgroup, ALKfus + tumors were significantly less likely to harbor APC, KRAS, and PIK3CA alterations, while additional differences in co-occurring genomic alterations were also observed (Table 6).

**Table 5.** Molecular characteristics of MSI-high CRC according to ALK fusion status.

	ALKfus+	ALKfus−	*P*-value
**Median TMB (IQR)**	45.63 (37.75-59.38)	45.86 (33.76-60.34)	1.000
**PD-L1 positive**	28.6%	41.2%	.707
***APC***	22.2%	40.1%	.782
***BRAF***	5.6%	48.3%	.010
***GATA6***	5.6%	0.4%	.593
***KMT2D***	72.2%	54.2%	.782
***KRAS***	5.6%	27.5%	.432
***NOTCH1***	5.6%	13.4%	.898
***PIK3CA***	0.0%	37.6%	.012
***RNF43***	65.5%	88.9%	.502
***SOX9***	27.8%	29.5%	1.000

**Table 6.** Molecular characteristics of MSS CRC according to ALK fusion status.

	ALKfus+	ALKfus−	*P*-value
**Median TMB (IQR)**	3.75 (2.41-6.25)	3.62 (1.25-5.00)	.858
**PD-L1 positive**	26.7%	13.1%	.123
***APC***	35.6%	80.7%	<.0001
***BRAF***	6.7%	8.4%	1.000
***GATA6***	6.7%	0.8%	.023
***KMT2D***	6.7%	1.2%	.046
***KRAS***	11.1%	49.7%	<.0001
***NOTCH1***	6.7%	0.7%	.015
***PIK3CA***	0.0%	17.5%	.002
***RNF43***	15.6%	3.2%	.003
***SOX9***	0.0%	10.2%	.041

## Discussion

We present the largest cohort of ALKfus + CRCs reported to date and characterize their distinct clinical and genomic features. Within this rare subset of CRC, we observed lower frequencies of canonical driver alterations and clinically actionable mutations, including *KRAS*, *APC*, *PIK3CA*, and *BRAF V600E*.^36–38^ This mutual exclusivity with established CRC oncogenic pathways is consistent with ALK fusions functioning as a primary oncogenic driver in these tumors.

We identified several recurrent ALK fusion partners, including previously described ALK fusions in CRC such as *CAD-ALK*, *EML4-ALK*, *SPTBN1-ALK*, *STRN-ALK*, and *DIAPH2-ALK*.^39^^,^^40^ We also detected *ATIC-ALK*, a fusion not previously reported in CRC but that has been described in ALCL and rarely in NSCLC, IMT, and glioma.^41–44^ Finally, we identified *ROCK2*, located on chromosome 2p25.1, as a novel fusion partner of ALK (2p23.2), consistent with an intrachromosomal rearrangement on chromosome 2. The predominant fusion partners were similar when stratified by MSI status. These recurrent and novel ALK fusion partners expand the spectrum of ALK rearrangements observed in CRC and underscore the molecular diversity of this rare disease subset.

ALK inhibitors have robust activity in ALKfus + NSCLC, ALCL, and IMT with reported response rates ranging from 50% to 85%.^6^ However, their use in other cancer types, including CRC, remains poorly defined, as the extremely low prevalence of ALK fusions limits the feasibility of prospective clinical trials.^6^ Nevertheless, published case reports and case series have reported responses to ALK-directed therapies in ALKfus + CRC.^21–24^ Together with the marked depletion of other clinically actionable alterations observed in our cohort, the reported clinical activity of ALK-directed therapies further supports comprehensive genomic profiling to identify patients with this rare but potentially targetable molecular subgroup of CRC.

Our analysis demonstrated marked enrichment of MSI-high disease in ALKfus + CRCs. This observation is consistent with prior reports that oncogenic gene fusions are enriched among MSI-high/dMMR CRCs.^45^^,^^46^ Structural variants and gene fusions are primarily driven by defective DNA double-strand break repair mechanisms, rather than by global point mutation-driven genomic instability.^31^^,^^47^ Nevertheless, prior analysis of fusion breakpoints in MSI-high CRC has implicated inefficient repair of microbiome-induced clustered oxidative DNA damage in the setting of a mismatch repair deficiency as a potential mechanism underlying fusion formation.^48^ Furthermore, clonality may have important implications for the biological and clinical significance of kinase fusions, and a recent study suggested that clonal kinase fusions are preferentially enriched in MSI-high CRC.^49^ Because fusion allele frequency data were not available in our cohort, we were unable to determine whether ALK fusions represented clonal or subclonal events, and future studies will be needed to clarify the evolutionary timing of ALK fusion acquisition in MSI-high CRC.

In addition to the enrichment of MSI-high disease, we also observed higher TMB and PD-L1 expression in ALKfus + CRCs. However, exploratory analyses stratified by MSI status suggested that these immune-associated features were largely driven by the overrepresentation of MSI-high tumors rather than ALK fusion status itself. In contrast, the depletion of canonical CRC driver alterations persisted after MSI stratification despite differences in the specific co-alteration patterns within the MSI-high and MSS subgroups, supporting the idea that ALK fusions represent an alternative oncogenic driver that is not solely explained by MSI-high enrichment.

The frequent co-occurrence of ALK fusions and MSI-high disease has important clinical implications. Both ALK-directed therapies and immune checkpoint inhibitors may be relevant therapeutic considerations for this patient population. However, the optimal integration and sequencing of these treatment approaches remain undefined. Reports of toxicity associated with concurrent administration of ALK inhibitors and immune checkpoint inhibitors in ALKfus + NSCLC suggest caution when considering combination approaches.^50^ Additional clinical investigation is needed to better define the role and sequencing of these therapies in patients with ALKfus + MSI-high CRC.

Our study has several limitations. First, the retrospective nature of this analysis and the lack of detailed treatment and outcome data preclude direct assessment of clinical responses to ALK-targeted therapies in ALKfus + CRC, as well as responses to immunotherapy among MSI-high tumors harboring ALK fusions. Second, the use of clinically submitted samples may introduce selection bias. Finally, functional validation of novel ALK fusion partners in CRC was limited, so we cannot definitively determine the oncogenic potential of the fusions identified.

In conclusion, ALK fusions define a rare but biologically distinct subset of CRC characterized by a unique genomic landscape, including frequent MSI-high disease and depletion of common CRC driver mutations. These findings support comprehensive genomic profiling to identify ALK fusions in CRC and highlight the potential role of ALK-directed therapies in this molecular subgroup. The frequent co-occurrence of MSI-high disease also raises important questions regarding the integration and sequencing of targeted therapy and immune checkpoint blockade that warrant further clinical investigation.

## Data Availability

The data that support the findings of this study were generated through comprehensive genomic profiling performed by Foundation Medicine. De-identified data may be available from the corresponding author and/or the data provider upon reasonable request and with appropriate data use agreements, subject to institutional and company policies.
